# Community-based rehabilitation interventions on quality of care for people with schizophrenia in China (CRISC): study protocol for a cluster-randomized controlled trial

**DOI:** 10.1186/s12888-023-04774-0

**Published:** 2023-05-12

**Authors:** Ruoxi Ding, Miaomiao Zhao, Yanshang Wang, Ming Wang, Dan Guo, Xiao Liu, Lei Wang, Wentao Wei, Wei Zhang, Ping He

**Affiliations:** 1 China Center for Health and Development Studies, Peking University, Beijing, China; 2 School of Public Health, Peking University, Beijing, China; 3 Shanghai Mental Health Center, Shanghai Jiao Tong University School of Medicine, Shanghai, China; 4 Center for Mental Health Management, China Hospital Development Institute, Shanghai Jiao Tong University, Shanghai, China; 5 Mental Health Branch, China Hospital Development Institute, Shanghai Jiao Tong University, Shanghai, China; 6 School of Management, Weifang Medical University, Weifang, Shandong province China; 7 Weifang Kuiwen District Medical care and Health Industry Development Center, Weifang, Shandong province China; 8 Weifang City Hanting District Gudi street Pozi hospital, Weifang, Shandong province China; 9 Weicheng District health comprehensive law enforcement brigade, Weifang, Shandong province China

## Abstract

**Background:**

International consensus shows that community-based rehabilitation (CBR) service is an effective way to improve functioning and negative symptoms and address the treatment gap for schizophrenia. Rigorous trials are needed in China to demonstrate effective and scalable CBR interventions to significantly improve outcomes for people with schizophrenia and to provide evidence of the economic benefits. The objectives of this trial are to examine the effectiveness of CBR as an adjunct to test the usual facility-cased care (FBC) in comparison to FBC alone in improving a range of outcomes in people with schizophrenia and their caregivers.

**Methods:**

This trial is a cluster randomized controlled trial design in China. The trial will be conducted at three districts of Weifang city, Shandong province. Eligible participants will be identified from the psychiatric management system where community-dwelling patients with schizophrenia have been registered. Participants will be recruited after providing informed consent. 18 sub-districts will be randomly allocated in a 1:1 ratio to facility-based care (FBC) plus CBR (intervention arm) or FBC alone (control arm). The structured CBR intervention will be delivered by trained psychiatric nurses or community health workers. We aim to recruit 264 participants. The primary outcomes include symptoms of schizophrenia, personal and social function, quality of life, family burden of caring, etc. The study will be conducted according to good ethical practice, data analysis and reporting guidelines.

**Discussion:**

If the hypothesized clinical benefit and cost-effectiveness of CBR intervention are confirmed, this trial will provide significant implications for policy makers and practitioners to scale up rehabilitation services, as well as for people with schizophrenia and their family to promote recovery and social inclusion, and to alleviate the burden of care.

**Trial registration:**

Chinese Clinical Trial Registry (ChiCTR2200066945). Registered December 22, 2022.

## Background

Schizophrenia is a major severe mental disorder that has profound adverse effects on individuals and poses huge disease burden to families [1]. It usually has an onset in early adulthood and is often accompanied with persistent or relapsing symptoms. According to the estimation of the Global Burden of Disease Study, globally, schizophrenia accounted for 6.51 million DALYs in 2017, representing 0.51% of the all-cause DALYs [2]. Despite its low prevalence, the mortality rate of people with schizophrenia is two to three times higher than that in the general population [3]. And most people with schizophrenia have difficulties with work and social participation. In China, 6 million individuals suffer from schizophrenia: 80% of them with moderate to severe disability; 40% of them have never received treatment [4, 5]. Such high risk of disability among schizophrenic patients and consequent adverse outcomes, which are likely due to extremely low access to psychiatric services, has made schizophrenia a priority public concern in recent years.

International consensus shows that community-based rehabilitation (CBR) service, as a broader supportive approach, is an effective way to improve functioning and negative symptoms and address the treatment gap for schizophrenia [6]. In particular, the WHO’s mhGAP guidelines and the third edition of the Word Bank’s Disease Control Priorities (DCP3) both recommended that community-based rehabilitations as an adjunct to antipsychotic treatment for schizophrenia [7]. The rehabilitation interventions, which can be implemented by trained community workers, are more needful in low- and middle-income countries due to the shortage of psychiatrists and psychiatric services [8]. These interventions have been proven to be effective and acceptable to address the broader social and livelihood needs of service users in some low- and middle- income countries (LMICs), such as India [9] and Ethiopia [10].

Community-based mental rehabilitation has received high-level attention from Chinese national policy makers recently. China’s first Mental Health Law, which was enacted in 2012 and enforced in 2013, requested to provide mental rehabilitation services to psychiatric patients. The Strategic Plan for Healthy China 2030 issued in 2016 clearly stated the mission of “equal access to rehabilitation services for all disabled persons”. Suggestions on Accelerating the Development of Community-based Rehabilitation Services among People with Mental Disorders issued in 2017, provided specific steps to implement and scale up community-based rehabilitations: 80% of counties (districts) can provide mental rehabilitation services and 60% of patients with mental disorders can receive these services in 2025.

However, there is a lack of high-quality study on how to effectively implement community-based rehabilitation interventions for schizophrenia, and to conduct the health economic evaluations for the interventions in China, which weakens evidence base for policymaking. Therefore, rigorous trials are needed in China to demonstrate effective and scalable interventions to significantly improve outcomes for people with schizophrenia and to create evidence of the economic benefits of these interventions, for use in advocacy with the government to establish national programs. This paper presents the protocol for community-based rehabilitation interventions on quality of care for people with schizophrenia in China (CRISC) project, which is a cluster randomized trial that evaluating the effectiveness of CBR intervention.

## Objectives

The objectives of the trial are: first, to examine the effectiveness of CBR as an adjunct to test the usual facility-cased care (FBC) in comparison to FBC alone in improving a range of outcomes in people with schizophrenia and their caregivers at 12 months and, second, to determine if the CBR intervention is cost effective in China.

The primary objectives of the trial are to determine whether the FBC + CBR intervention will be more effective than FBC alone in:

i) reducing symptoms of schizophrenia at 12 months;
ii) improving physical function and quality of life for people with schizophrenia over 12 months, and.
iii) reducing the burden of caring for caregivers of people with schizophrenia at 12 months.

The secondary objectives are to determine whether, FBC + CBR will be superior to FBC alone, in:

i) improving adherence to antipsychotic treatment at 6 months and 12 months,
ii) reducing the hospital readmission rate at 6 months and 12 months for people with schizophrenia, and.
iii) improving the knowledge about schizophrenia of people with schizophrenia and their caregiver at 6 months and 12 months.

## Methods

### Study design

**Fig. 1:**
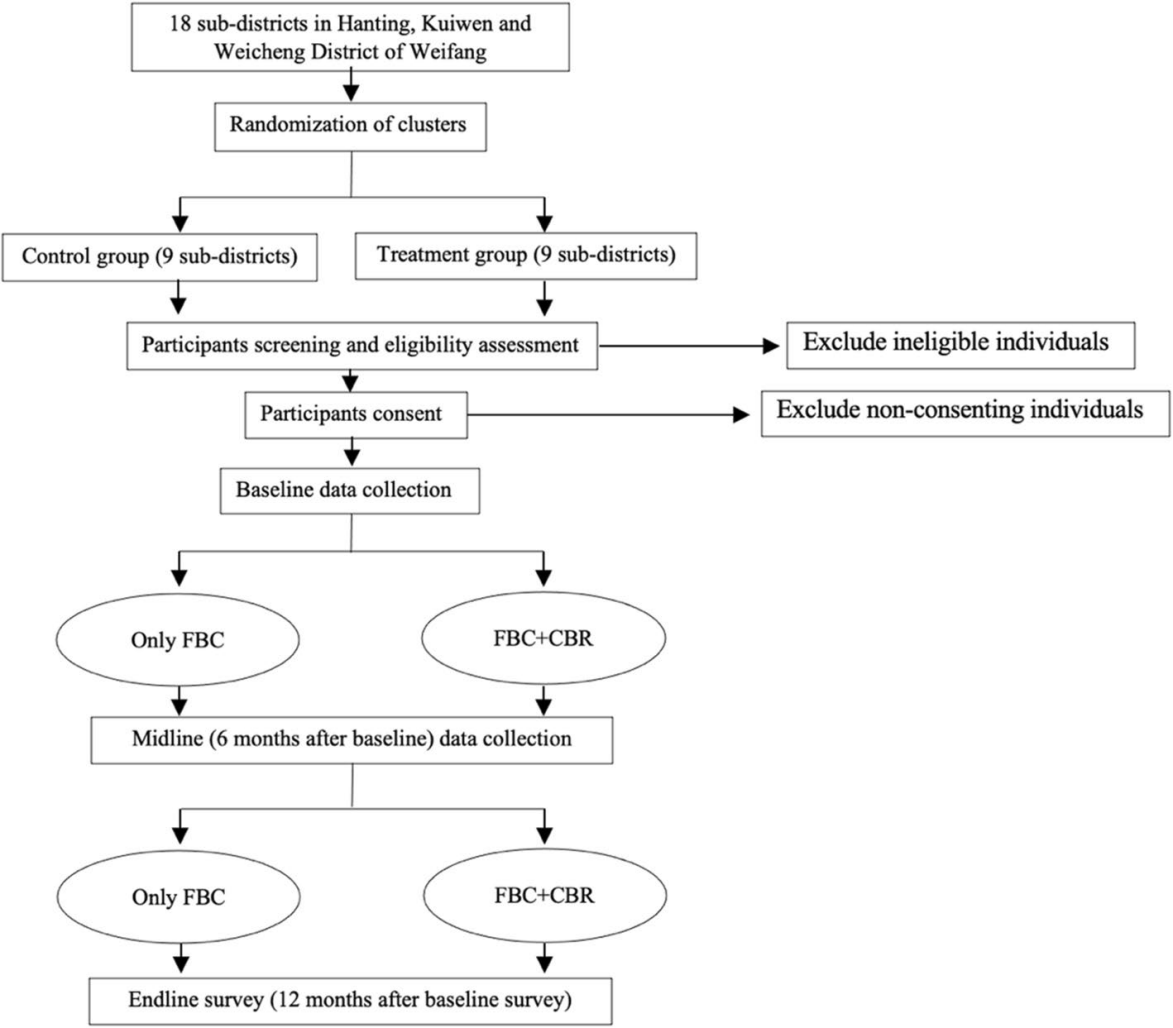
CRISC flow chart

The design is a cluster randomized controlled trial with sub-district as the unit of randomization. There are mainly three reasons for choosing cluster randomization as the method of randomization: (1) CBR was provided as group intervention at community healthcare, which exclude the possibility of randomization at individual level; (2) randomization at sub-district will be more economical and less time-consuming for both provider and participants; (3) The acceptability will be maximized, and the possibility of contamination could be minimized if all participants in a sub-district are allocated to either intervention or control arms. The study flow chart is presented in Fig. 1. Eighteen sub-districts from three district in Weifang, Shandong province were included for the trial, 9 will be randomly allocated to the intervention arm (FBC plus CBR) and 9 randomly allocated to the control arm (FBC alone). In total, 334 participant dyads (patients and their caregivers) will be recruited, with 18.6 participant dyads on average recruited for each sub-district.

### Setting

The trial will take place in Hanting, Kuiwen and Weicheng district, Weifang city, Shandong province in China. The three districts have a population of 1,470,000 persons in 23 sub-districts. Six sub-districts in each district were chosen, yielding to 18 sub-districts in the trial. There is a community health center in each sub-district that covering the facility-based care of local people with severe psychiatric disorders including schizophrenia. The number of registered schizophrenia patients of each sub-district ranges from 52 to 138, with 12 to 25 patients are planned to recruit.

### Participant inclusion criteria

The inclusion criteria for participating in the trial are: (1) Be aged 18–59 years; (2) Have a primary diagnosis of schizophrenia by psychiatrists according to ICD-10 DCR criteria; (3) Have had the an illness duration of at least 12 months; (4) Be residing within the sub-district for the next 12 months; (5) Have never been diagnosed with severe physical conditions, drug or alcohol dependence, organic brain disease, learning disability or hearing impairment; (6) Have a primary caregiver who is willing to participate in the study; and (7) being in stable conditions and community -dwelling. The exclusion criteria are: (1) being hospitalized due to schizophrenia at the time of recruitment; (2) being illiterate or physically incapable of text or verbal communication.

### Participant flow

#### Participant identification

In China, most of the community-dwelling patients with schizophrenia who have been diagnosed in hospitals or primary care services are registered in the National Information System for Psychosis of China. The recruitment processes of this trial are as follows: Primary care physicians from 18 sub-districts of Hanting, Kuiwen and Weicheng districts, identify the potential participants who met the inclusion criteria from their local registered community-dwelling patients with schizophrenia. Eligible patients will be invited to join the trial by primary care physicians or nurses. They will be given information about the trial and the study procedure in a way appropriate to the participants’ literacy level by telephone or face-to face. Those who consent (or, for those without decision-making capacity, the caregiver will consent on their behalf) to participate will go to community health care center to sign the informed consent and complete baseline data collection. Within each sub-district, the recruitment will continue until approximately 20% of community-dwelling patients have been recruited.

#### Allocation

The randomization processes of sub-districts to intervention and control arms are as follows: (1) The sociodemographic characteristics and specific information of patients with schizophrenia of each sub-district will be obtained from the information system [11]; (2) A pairwise matching work will be conducted to identify 9 pairs of matched sub-district that share similar characteristics (number of population, prevalence of schizophrenia, average income per capita, etc.); (3) Within each pair, one sub-district will be randomly assigned into intervention arm, and the remaining one will be assigned into control arm, yielding to 9 sub-districts in intervention arm that providing FBC + CBR, and 9 sub-districts in control arm with FBC only. The sub-district allocation list will be kept securely on a password-protected document by the trial coordinator. To reduce selection bias, the potential eligible participants will not be informed about the allocation of their sub-district until they have consented to participate and baseline data of all participants have been collected. Recruitment and participant flow will be closely monitored by the trial coordinator.

### Intervention

#### Facility-based care

FBC will be available to all participants in this trial. Facility-based care is the care usually provided by mental health practitioners for persons with schizophrenia and their families. No guidelines or protocols are available for FBC as this is more representative of usual care in China. Psychiatrists from general or psychiatric hospitals prescribed antipsychotic medications and provide consultation, including specific information about the illness, encouragement on adherence and discussion about other specific concerns. The frequency of FBC was once per month or more, which will be determined by clinical need.

#### Community-based rehabilitation

##### Intervention development

The CBR intervention was developed through four steps that employing a range of qualitative and participatory methodologies. First, a literature review of rehabilitation and psychosocial intervention for patients with schizophrenia was conducted, resources from the World Health Organization (WHO), The Community care for People with Schizophrenia in India (COPSI) [12], Community-based Rehabilitation Intervention for people with Schizophrenia in Ethiopia (RISE) [10], etc. were reviewed to identify potential components of CBR and the likely effectiveness. Second, a scoping workshop involving psychiatrists, psychiatric nurses, community health workers and local healthcare managers was implemented to analyze the feasibility of CBR components and delivery mechanism. Third, we arranged several in-depth interviews and focus group discussions with people with schizophrenia and their caregiver and primary healthcare workers to understand the unmet needs and to evaluate the acceptability of CBR. Finally, a workshop involving CRISC director and coordinators, psychiatrists, psychiatric nursers and community health workers (potential CBR worker), local healthcare administrator and manager was conducted to synthesize the findings from step 1–3, based on which the detailed content of the intervention and the CBR training materials was developed.

Guided by previous evidence, the components of the intervention package are: 1) Structured psycho-educational information to improve the understanding of schizophrenia and address experience of illness-related stigma and discrimination; 2)proactive management of the illness to reduce medication non-adherence and improve prevention and identification of symptoms and relapse; 3) specific training strategies to improve daily life and social skills; 4) health promotion strategies to improve participants’ physical health status; 5)developing personalized rehabilitation plan to facilitate the supportive framework for participants’ further recovery and social integration.

##### CBR worker recruitment and training

Nineteen CBR workers were recruited from mental health center of the three districts in Weifang city according to the following criteria: (1) at least 12 years of schooling, (2) resident in Weifang city, (3) one-year-or-above working experience related to psychiatry or psychology and (4) interest in community work. CBR training was delivered by multiple sessions at different time (time before delivering new module) to ensure that each intervention module was fully learned and will be smoothly implemented. A intervention manual will be provided for each CBR worker. The competency of CBR workers will then be assessed using role-plays and patient vignettes.

##### CBR delivery

The CBR intervention will be delivered by group at community health centers. Two CBR workers will be attached to each sub-district and have one group of 10–20 people with schizophrenia. CBR delivery will commence immediately after recruitment into the trial. A total of 18 group intervention sessions will be implemented within the following 12 months, and each group intervention session will last 90–120 min. The intervention is delivered in two phases (Table 1): In phase 1 (intensive engagement phase), 12 intervention sessions will be delivered fortnightly during the first 6 months. The content of the intervention includes a series of modules such as developing a positive therapeutic alliance, understanding schizophrenia, adherence management, etc. (Table 1). In phase 2 (stabilizing phase), 6 intervention sessions will be delivered monthly during the last 6 months. Intervention modules in this phase are similar with phase 1 to reinforce the progress, and the content will include more in-depth discussion and address unmet needs from the first phase. In this phase, a personal rehabilitation plan for each participant will be generated to help their continued rehabilitation at home.

The content and process of the phase specific delivery of the intervention has been manualized. CBR workers will be closely supervised at each site by psychiatrists who will work as designated intervention coordinators. They will coordinate the overall delivery of the intervention and assure the quality and fidelity of the intervention at the site. The treating psychiatrists will provide clinical leadership for the community care teams and ongoing supervision to maintain safety and quality standards.

In total, we anticipate that each participant in the intervention arm will receive a maximum of 18 group-based rehabilitation sessions during the 12 months of the intervention. In evaluating the intervention, a minimum adequate number of 12 sessions attended over 12 months was set according to priori definitions.

**Table 1:** CRISC community-based rehabilitation (CBR) intervention outline

phase	months	frequency	Objectives	CBR Modules	CBR activity
Phase 1 (intensive engagement)	1–6	fortnightly	• developing a positive therapeutic alliance • needs assessment • improving knowledge of illness • improving medication adherence • addressing familial and social difficulties	• understanding schizophrenia • psychoeducation • adherence management • physical health promotion • life skill training • social skill training • symptoms management	• warm-up exercises (15 min) • introduction & ice-breaking activity (20 min, for the first session) • sharing experience of past two weeks (30 min) • Module activity (30–40 min) • question and discussion (20 min) • assignment for next two weeks (10 min)
Phase 2 (stabilizing)	7–12	monthly	• addressing the unmet or partially met needs from the first phase • reinforcing knowledge of illness • reinforcing medication adherence • improving self-care skills • improving social interactions within and outside of the family • Generating a rehabilitation and relapse prevention plan	• psychoeducation • adherence management • physical health promotion • life skill training • social skill training • symptoms management • personal rehabilitation planning • prepare for termination	• warm-up exercises (15 min) • sharing experience of past two weeks (30 min) • Module activity (30–40 min) • question and discussion (20 min) • making rehabilitation plan (20 min) • assignment for next two weeks (10 min) • CBR summary & energetic participants awards (20 min, for the last session)

### Outcome assessment

For the primary outcome assessments, we will record the following indicators as intermediate outcome for each participant at baseline, intermediate (6 months after initiating intervention) and endline (12 months after initiating intervention) survey:

- Symptoms of schizophrenia will be measured using the Positive and Negative Syndrome Scale (PANSS) [13].
- Personal and social function will be measured by Personal and Social Performance Scale (PSP) [14, 15].
- Antipsychotic adherence will be assessed using Medication Adherence Rating Scale (MARS) [16].
- Knowledge of illness will be measured using the Knowledge About Schizophrenia Test (KAST) [17] for each participant and their caregiver.
- Hospital readmission records will be extracted from electronic medical records using participants’ national identification number.

We will also be assessed the following indicators as long-term outcome for each participant at baseline and endline (12 months after initiating intervention) survey:

- Physical function will be measured using Activity of Daily Living Scale (ADL) and Instrumental Activity of Daily Living Scale (IADL).
- Social support of participants will be measured using Perceived Social Support Scale (PSSS) [18].
- Quality of life of participants will be assessed using the EuroQoL five-dimensional instrument (EQ-5D) and the Schizophrenia Quality of Life Scale (SQLS) [19].
- The family burden of caring will be measured through participants’ caregiver interview using Burden Assessment Scale for families of the seriously mentally ill (BAS) [20].

For the secondary outcome assessment, we will calculate the cost of schizophrenia by combining the self-reported cost from participants or their caregivers and the electronic medical records. The direct cost includes inpatient during the last year and outpatient cost during the last month, which will be extracted from the electronic medical records, and self-medication cost reported by participants or their caregivers. The indirect cost includes loss of wages of caregiver, fees of transportation, accommodation and meals due to accompanying and care, and other cost during the inpatient and outpatient service utilization. All the indirect costs were self-reported by participants or their caregivers. The secondary outcome will be assessed at baseline, intermediate and endline survey. Table 2 describe the details of each outcome measure in this study.

Additionally, participants’ and their primary caregivers’ sociodemographic information, participants’ health behavior, comorbid mental and physical disorder will be recorded during the baseline survey. Participants’ family history of mental disorders, history of schizophrenia, which include the year of the first episode, current and past use of antipsychotic medication will be extracted from information system, in which all the participants were registered and recorded.

Primary care physicians will collect all data except for symptoms of illness and personal and social function. We choose primary care physicians as data collector instead of other external personnel for the following reasons: (1) typically, the primary care physicians were the doctors that in charge of the community-dwelling people with schizophrenia in their sub-district, and thus they could directly extracted the illness record of participants (after obtained the informed consent), and reduce the survey duration to prevent the occurrence of negative emotions of participants; (2) the primary care physicians were quite familiar with the people with schizophrenia in their sub-district, which may reduce the difficulties during the survey since a substantial proportion of participants have stigma issue and are reluctant to answer questions from strangers. The primary care physicians will be trained by 3 two-hour group sessions in a structured manner to ensure adequate inter-rater reliability for all scale items across the sites. The symptoms of schizophrenia and personal and social function will be assessed by psychiatrists from the mental health center of each district. A 5-hours training session of PANSS and PSP scale were provided by experts in psychiatry from Peking University Sixth hospital. All the trainees will be asked to take an examination to assess the symptom and personal and social function of one patient with schizophrenia by video. To ensure the adequate inter-rater reliability, only those who pass the exam will be qualified for the PANSS and PSP assessment in the baseline, intermediate and endline survey. Participants’ data will be either extraction from information system or collected by face-to-face interview at community health center. For participants who do not go to community health center after three invitations, a home visit will be conducted by interviewer for data collection. The survey and assessment forms will be finished by Wenjuanxing, a Chinese online platform for survey, assessment and voting.

To maintain blinding of these assessments, the primary care physicians and psychiatrists who administered the outcome measures will be independent of the CBR intervention. The primary care physicians were unable to be blinded to the allocation of treatment since they were the coordinator to contact the participants from their local sub-districts. The psychiatrists, who will assess the main primary outcomes (symptoms of schizophrenia and personal and social function), will be blinded to the allocation of treatment. Since neither the participant nor the family caregivers will be blind to their allocation status at the time of the 6 and 12 month interviews, we intend to minimize the chances of unmasking during the outcome assessments by instituting the following measures: (1) the intervention team and data collection teams at the sites will not have any interactions during the trial; (2) the data collection team are told that they are evaluating two interventions that is unknown about which one is better; (3) orient the participants and their caregivers prior to each assessment that they should not disclose whether or not they are attending CBR.

**Table 2:** Summary of outcome measures

Outcomes (Intermediate outcome)	Interviewees	Assessment scale	Month 0 (Baseline)	Month 6 (Intermediate)	Month 12 (Endline)
**Primary outcome**					
* Intermediate outcome*					
Symptoms of schizophrenia	Participants	Positive and Negative Syndrome Scale (PANSS)	√	√	√
Personal and social function	Participants	Personal and Social Performance Scale (PSP)	√	√	√
Antipsychotic adherence	Participants	Medication Adherence Rating Scale (MARS)	√	√	√
Knowledge of illness	Participants & primary caregiver	the Knowledge About Schizophrenia Test (KAST)	√	√	√
Hospital readmission	Participants	Electronic medical records	√	√	√
* Long-term outcomes*					
Physical function	Participants	Activity of Daily Living Scale (ADL) & Instrumental Activity of Daily Living Scale (IADL)	√		√
Social support	Participants	Perceived Social Support Scale (PSSS)	√		√
Quality of life	Participants	the EuroQoL five-dimensional instrument (EQ-5D) & the Schizophrenia Quality of Life Scale (SQLS)	√		√
The family burden of caring	Primary caregiver	Burden Assessment Scale for families of the seriously mentally ill (BAS)	√		√
**Secondary outcome** Cost of illness	Participants/ Primary caregivers	Electronic medical records & self-reported self-medication cost, loss of wages of caregiver, fees of transportation, accommodation, and meals due to accompanying and care and other cost during the inpatient and outpatient service utilization	√	√	√

### Quality assurance and fidelity management

To ensure that the CBR intervention is of adequate fidelity, each district will be assigned a coordinator to monitor the delivery of CBR sessions in the three sub-districts from intervention arm every month. The intervention coordinators will record and rate the key contents of the CBR sessions such as interactions between CBR workers and participants, the ways of module presentation, etc., according to a specially designed rating scale. Regular meetings will be held to provide feedback for improving the delivery of the intervention to all CBR workers to maintain quality benchmarks of the trial. Any significant divergence from the intervention manual will lead to suitable corrective action to harmonize the intervention across the sites.

To ensure the adequate quality of outcome measurements in the baseline, intermediate and endline survey, three procedures of supervision will be implemented. First, all the interviews, including both the part of primary care physicians and will be sound recorded (after obtaining the consent of participants), the records will be directly sent to team researchers and experts in psychiatry (for symptoms and function assessment) every day for quality control purpose. Any divergence from the protocol norms will lead to communication with data collectors for correction, and significant errors will lead to re-interview for outcome measurements. Second, each district will be assigned a coordinator to monitor 20% interviews of every sub-district through randomly onsite visits. The coordinator will provide feedback based on a specially designed assessment form to the team researchers. Third, researchers will also conduct casual inspection to check the quality of 10% of the finished questionnaire and assessment through the online platform. The submitted questionnaire will be sent back to data collector for re-check if any illogical error was found. A weekly group meeting with data collectors, researchers and experts in psychiatry will be held to ensure the interview norms and to achieve the overall quality benchmarks.

### Statistical issue

#### Sample size estimation

Based on the data from the earlier random controlled trials [9, 21], it was assumed that the participants in the intervention arm will have 20% reduction on the PANSS total score mean reduction after the 12-months CBR, and there was no change observed for participants in the control arm. The intra-class correlation within the three districts was set at 0.05 and alpha was set as 0.05. Based on methods from previous literature [22], a sample size of 110 participants in both intervention arm control arm was calculated for this trial, which will give us 90% power to detect the difference. The required sample size of 220 was increased to 264 to allow for 20% attrition. The total number of participants will be divided slightly unequally between sub-districts for reasons of feasibility.

#### Data management and analysis

Data collection and management will follow Good Clinical Practice (GCP) guidelines and Standard Operating Procedure. Each participant will be assigned a unique ID so that their reported data could be identified. Data cleaning will follow standard procedure, and all the changes will be documented. Findings of this trial will be reported according to the revised CONSORT guidelines [23]. Stata 16.0 will be used for quantitative analyses. Data will be password-protected and only accessible for authorized personnel of the research team. In accordance with good trial practices, data and all related documentation will be stored for a minimum of 7 years after the completion of the trial.

All statistical analyses were performed using STATA version 16.0 for Mac (Stata Corp, College Station, TX, USA). Descriptive statistics will be applied to summarize the socio-demographic and clinical characteristics, as well as the outcome measures for all trial participants at baseline, intermediate and endline. Participants who are loss-to-follow-up will be compared using chi-square tests and t tests to assess the potential bias. The primary outcome analysis will be masked until the analyses is finalized and approved by all researchers. Adequate intervention will be defined as having received a minimum of 12 group sessions. We will not define adequate FBC for the control arm a priori. CBR worker’s characteristics such as educational background, post-training competency and relative working experience will also be described.

Data analysis will be conducted under intention-to-treat assumption. The primary outcome----12-month PANSS total score, adjusted for PANSS scores at baseline and site, and including clustering effects of CBR workers, will be used for examining the comparative effects of intervention and control arm. Personal and social function (PSP score), antipsychotic adherence (MARS score), knowledge of schizophrenia (KAST score) and hospital readmission will be similarly analyzed. A longitudinal analysis including both 6- and 12-month outcomes will also be performed using a GEE or random effects model including an interaction term of time and treatment allowing for modelling changes over time. Long-term outcomes (ADL & IADL, PSSS, EQ-5D, SQLS and BAS score) at 12 months will be analyzed in a similar way to the primary outcome. We will also perform residual analysis to check the data distribution and to detect outliers. Multiple imputation methods will be employed for participants with missing outcome variables.

Several sensitivity analyses are planned to be carried out. First, a complete case analysis and a complier average causal effect will be conducted. Second, a dose-response relationship between adherence to CBR intervention (number of group sessions attended) and the intermediate and long-term outcomes will be examined. Third, we will also conduct data analyses that including received quantity of different CBR modules to identify the active components. Furthermore, regression models will also be re-estimated to include possible confounders such as characteristics of CBR workers and participant characteristics that differ at baseline.

Exploratory subgroup analyses by gender, urban-rural residence, baseline symptom severity and antipsychotic adherence will be completed, although the power for these analyses has not been specifically allowed for.

#### Cost-effectiveness analysis

Direct costs of the CBR intervention will be estimated by deriving a monetary value based on actual cost. Cost of illness, as the secondary outcome will be computed based on electronic medical records & self-reported data, and then compared at intermediate line and endline. Cost-effectiveness will be assessed by calculating the incremental cost-effectiveness ratios, which will show the extra cost incurred to produce a unit improvement in symptoms, personal and social function, as well as QALYs gained at the endline. To explore the uncertainty around the estimates, cost-effectiveness acceptability curves will be derived to provide the probability of advantages for the FBC combined CBR at a range of ‘willingness to pay’ threshold levels.

### Trial management and monitoring

The CRISC study will be conducted according to the Good Clinical Practice (GCP) guidelines recommended for conducting clustered randomized controlled trials. A data safety team will be organized to ensure the privacy and safety of trial participants by both onsite visiting and backstage management. This study will also assign a dedicated coordinator to monitor and coordinate the recruitment and progress of the trial. All protocol violations will be recorded in trial report.

### Ethical considerations

Participants’ rights are fully protected in accordance with good practice ethical obligations. during every stage of the trial. Informed consent to participate will be designed in compliance with the GCP guidelines and Helsinki Declaration [24]. Informed consent form will be obtained from every participant, and for those who are symptomatic or have difficulties to read, the consent procedure will be carried out using non-technical language and other possible manners to enhance the intake of information.

Each participant and their primary caregiver will be assigned a unique ID, and their personal identification items will be removed and kept as a separate file with restricted password to protect the confidentiality. All study data will be collected and collated using only the ID. Formal ethical approval for conducting the trial has been obtained from Institutional Review Board (IRB) at Peking University (Approval number IRB00001052-21170). Any adverse event reports will be submitted to IRB, which will approve any protocol modifications as necessary.

## Discussion

Schizophrenia poses a huge disability and societal burden in low- and middle-income countries (LMICs). The importance and urgency of community-based rehabilitation services has been recognized by Chinese government. Building upon the feasibility, acceptability of CBR programs in previous observational study and random-controlled trial from other LMICs, the CRISC study is designed to provide high-quality evidence about the effectiveness and the economic implications of the CBR intervention among people with schizophrenia in China. If the hypothesized clinical benefit and cost-effectiveness of CBR intervention are confirmed, this trial will provide significant implications for policy makers and practitioners to scale up rehabilitation services, as well as for people with schizophrenia and their family to promote recovery and social inclusion, and also to alleviate the burden of care.

## Data Availability

Not applicable.
